# Curcumin Diglutaric Acid, a Prodrug of Curcumin Reduces Pain Hypersensitivity in Chronic Constriction Injury of Sciatic Nerve Induced-Neuropathy in Mice

**DOI:** 10.3390/ph13090212

**Published:** 2020-08-27

**Authors:** Thanchanok Limcharoen, Peththa Wadu Dasuni Wasana, Hasriadi, Chawanphat Muangnoi, Opa Vajragupta, Pornchai Rojsitthisak, Pasarapa Towiwat

**Affiliations:** 1 Inter-Department Program of Pharmacology, Graduate School, Chulalongkorn University, Bangkok 10330, Thailand; 6087149020@student.chula.ac.th; 2 Pharmaceutical Sciences and Technology Program, Faculty of Pharmaceutical Sciences, Chulalongkorn University, Bangkok 10330, Thailand; dasuniwasana@ahs.ruh.ac.lk (P.W.D.W.); adhiehasri@gmail.com (H.); 3 Institute of Nutrition, Mahidol University, Salaya 73170, Thailand; chawanphat.mua@mahidol.ac.th; 4 Research Affairs, Faculty of Pharmaceutical Sciences, Chulalongkorn University, Bangkok 10330, Thailand; opa.vaj@mahidol.ac.th; 5 Department of Food and Pharmaceutical Chemistry, Faculty of Pharmaceutical Sciences, Chulalongkorn University, Bangkok 10330, Thailand; pornchai.r@chula.ac.th; 6 Natural Products for Ageing and Chronic Diseases Research Unit, Chulalongkorn University, Bangkok 10330, Thailand; 7 Department of Pharmacology and Physiology, Faculty of Pharmaceutical Sciences, Chulalongkorn University, Bangkok 10330, Thailand

**Keywords:** neuropathic pain, chronic constriction injury, curcumin diglutaric acid, mechanical allodynia, thermal hyperalgesia

## Abstract

The drug treatment for neuropathic pain remains a challenge due to poor efficacy and patient satisfaction. Curcumin has been reported to alleviate neuropathic pain, but its clinical application is hindered by its low solubility and poor oral bioavailability. Curcumin diglutaric acid (CurDG) is a curcumin prodrug with improved water solubility and in vivo antinociceptive effects. In this study, we investigated the anti-inflammatory mechanisms underlying the analgesic effect of CurDG in the chronic constriction injury (CCI)-induced neuropathy mouse model. Repeated oral administration of CurDG at a low dose equivalent to 25 mg/kg/day produced a significant analgesic effect in this model, both anti-allodynic activity and anti-hyperalgesic activity appearing at day 3 and persisting until day 14 post-CCI surgery (*p* < 0.001) while having no significant effect on the motor performance. Moreover, the repeated administration of CurDG diminished the increased levels of the pro-inflammatory cytokines: TNF-α and IL-6 in the sciatic nerve and the spinal cord at the lowest tested dose (equimolar to 25 mg/kg curcumin). This study provided pre-clinical evidence to substantiate the potential of pursuing the development of CurDG as an analgesic agent for the treatment of neuropathic pain.

## 1. Introduction

Neuropathic pain is characterized by abnormal signaling of pain pathways arising from injury or malfunction in the central or peripheral nervous systems [1]. Major symptoms of neuropathic pain include hyperalgesia, allodynia, and spontaneous pain. Hyperalgesia and allodynia are the elevated sensitivity to painful and non-painful stimuli, respectively. Since prolonged neuropathic pain leads to major suffering, lower quality of life, and disability, it is considered as a major factor contributing to the global burden of disease. Current pharmacotherapy for neuropathic pain includes tricyclic antidepressants, serotonin-norepinephrine reuptake inhibitors, calcium channel α-2-δ ligands, and opioids. However, the complexity of this disease and also the side effects and low efficacy in pain relief associated with the currently available pharmacological agents create an opportunity to develop effective treatments to relieve neuropathic pain [2,3].

A growing body of evidence indicates the relevance of immune responses in neuropathic pain. It has been reported that in nerve injury, peripheral immune cells release pro-inflammatory mediators and activate the peripheral nerves. Meanwhile, in the spinal cord, pro-inflammatory mediators, including cytokines, chemokines, growth factors, and gliotransmitters, are significantly increased as a response to peripheral nerve injury. TNF-α and IL-6 are involved not only in peripheral sensitization but also in central sensitization in the spinal cord [4,5]. However, most of the existing drugs for neuropathic pain only target neuronal cells, whereas the non-neuronal cells, such as peripheral immune cells and spinal glia, also contribute to the pathogenesis of neuropathic pain [6,7]. Therefore, finding new compounds that can modulate peripheral immune cells and spinal glia to dampen pro-inflammatory cytokines, such as TNF-α and IL-6, will be a promising therapeutic strategy for neuropathic pain treatment.

Numerous studies have suggested the usefulness of herbal medicines in the management of neuropathic pain [8]. In particular, curcumin is a phenolic compound derived from Curcuma longa (turmeric), which exhibits various pharmacological properties in pain relief, such as anti-nociceptive, anti-inflammatory, and neuropathic pain treatment, without or minimum toxicity with both short- and long-term usage [9]. Specifically, the analgesic activity of curcumin has been demonstrated in several models of neuropathic pain, such as peripheral nerve injury [10], diabetic neuropathy [11], HIV-neuropathy [12], and chemotherapy-induced peripheral neuropathy (CIPN) [13]. Moreover, curcumin has been shown to induce epigenetic modifications associated with pain [14]. However, curcumin possesses poor water solubility and rapid metabolism, resulting in poor oral bioavailability. Therefore, several approaches and formulation strategies have been used to improve the oral bioavailability of curcumin, such as nanoformulation, adjuvants, and prodrugs [9,15,16,17,18]. The prodrug approach for improving physicochemical and pharmacokinetic properties of curcumin includes conjugating curcumin with polymers [19], sugars [20], amino acids [21], retinoic acid [22], fatty acids [23], and dicarboxylic acids, such as succinic acid [24,25] and diglutaric acid [26].

**Figure 1:**
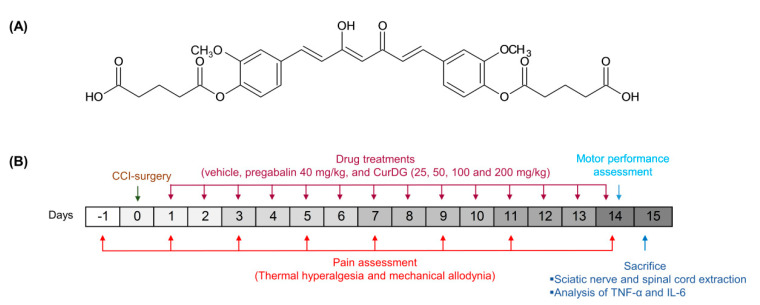
(**A**) Chemical structure of curcumin diglutaric acid (CurDG), and (**B**) the schematic illustration of experimental design.

Curcumin diglutaric acid (CurDG) (Figure 1A) has been developed as a potentially more soluble prodrug of curcumin by conjugating glutaric acid to curcumin via an ester linkage. Our preliminary studies showed that CurDG exhibited higher anti-nociceptive activity in the hot-plate analgesia test in mice compared to curcumin. The solubility of CurDG in water (7.48 μg/mL) was found to be 100 times higher than that of curcumin (0.07 μg/mL). Moreover, curcumin and CurDG were less soluble in both 0.1 M HCl and acetate buffer (pH 4.5) at 37 °C, while it was highly solubilized (1.43 μg/mL) in phosphate buffer (pH 6.8) compared to curcumin (0.025 μg/mL). In addition, the hydrolysis rate of CurDG at physiological pH 7.4 (K*_obs_* 2.62 h^−1^) was observed to be greater than that at low pH 1.2 and pH 4.5 (*K_obs_* 0.048 and 0.033 h^−1^, respectively), suggesting the potential of CurDG to prolong the degradation time of the released curcumin, which enables the gradual absorption of CurDG through the cell membrane till the rapid release of curcumin in the plasma. The *K_obs_* and half-life in human plasma for CurDG hydrolysis were 5.83 h^−1^ and 0.12 h, respectively, and the prodrug was fully converted to curcumin in human plasma within 2 h [26]. Therefore, it is of interest to further develop CurDG as a potential compound for the treatment of neuropathic pain. In this study, we investigated the anti-inflammatory mechanisms underlying the analgesic effects of CurDG in the chronic constriction injury (CCI) of sciatic nerve-induced neuropathy in mice (Figure 1B).

## 2. Results

### 2.1. Anti-Allodynia Activity

**Figure 2:**
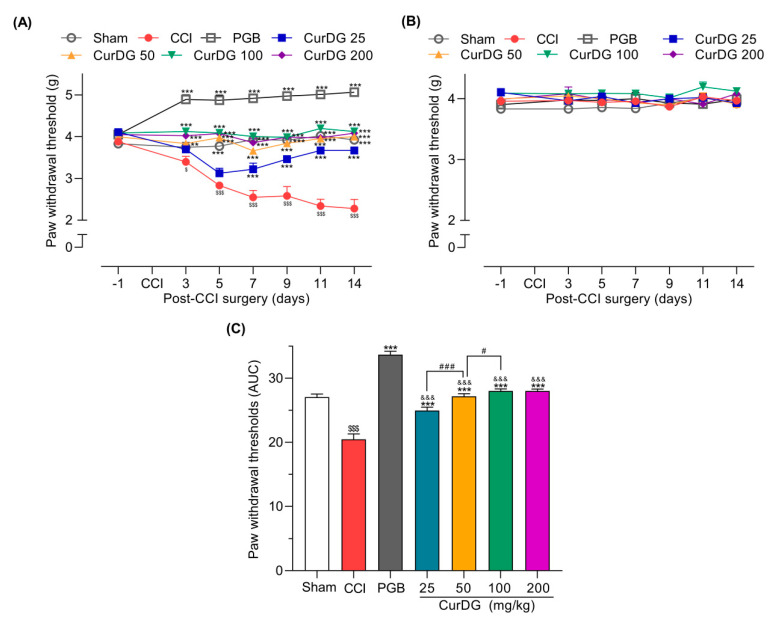
Anti-allodynic effect of CurDG at 25, 50, 100, and 200 mg/kg on the paw withdrawal thresholds (PWT) of (**A**) ipsilateral and (**B**) contralateral paws using von Frey test in chronic constriction injury (CCI)-mice (*n* = 8 mice/group). (**C**) Quantification of CurDG analgesic effects using the area under the curve (AUC) of PWT changes over time. ** and *** indicate a significant difference compared to the vehicle-treated group at the levels of *p* < 0.01 and *p* < 0.001, respectively. ^$^ *p* < 0.01 and ^$$$^ *p* < 0.001 compared to sham group, ^&&&^ *p* < 0.001 compared to pregabalin-treated group (PGB) and ^#^ *p* < 0.05 and ^###^ *p* < 0.001 compared between successive doses of CurDG.

As shown in Figure 2A, 3 days post-CCI surgery, mice displayed a significant reduction in paw withdrawal threshold (PWT) compared to the baseline (*p* < 0.05), which gradually declined from 3.88 ± 0.026 g to 2.28 ± 0.218 g over 14 days. Further, a significant improvement in the PWT compared to the control was observed on day 3 post-CCI in mice administered 50, 100, and 200 CurDG, wherein the mice administered 25 CurDG showed significant analgesia starting from day 7. In addition, the maximum analgesic effect of each dose was observed on day 14. As illustrated in Figure 2B, the PWT of contralateral paw had no effect either from the surgery or CurDG treatments. The area under the curve for the PWT changes over time (Figure 2C) also demonstrated that CurDG treatment at all dose levels had a better overall effect than vehicle treatment (*p* < 0.05). Moreover, pregabalin (40 mg/kg) exhibited a higher efficacy where the anti-mechanical allodynic effect of CurDG at all doses was significantly lower than that of pregabalin (*p* < 0.001).

### 2.2. Anti-Hyperalgesia Activity

**Figure 3:**
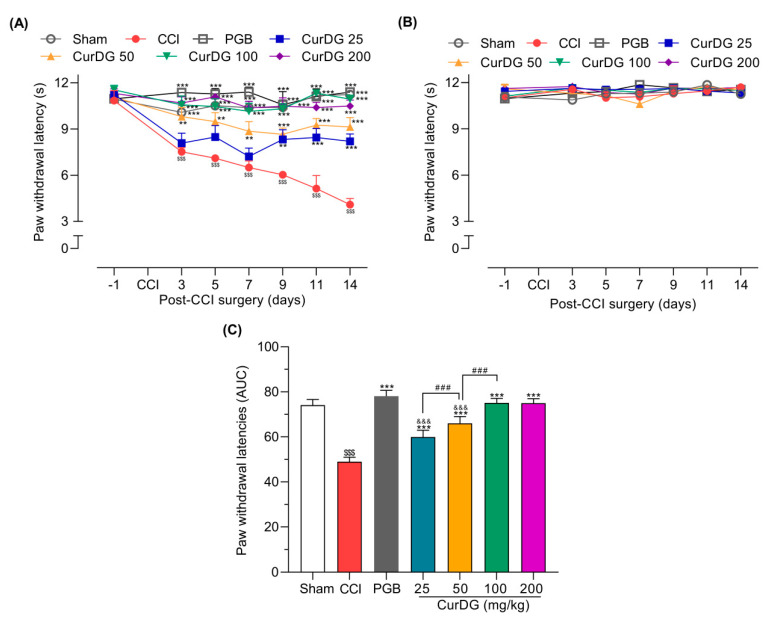
Anti-hyperalgesic effect of CurDG at 25–200 mg/kg on the (**A**) ipsilateral and (**B**) contralateral paw withdrawal latency (PWL) using the plantar test in CCI-mice (*n* = 8 mice/group). (**C**) The dose-AUC of ipsilateral PWL over the time course of drug administration. ** and *** indicate the statistical significance compared to the vehicle-treated group at *p* < 0.01 and *p* < 0.001, respectively. ^$$$^ *p* < 0.001 compared to sham group, ^&&&^ *p* < 0.001 compared to the pregabalin-treated group (PGB), and ^###^ *p* < 0.001 compared between successive doses of CurDG.

After the CCI surgery, mice showed a gradual and significant reduction in paw withdrawal latency (PWL) in the ipsilateral paw from 10.85 ± 0.64 s to 4.09 ± 0.39 s (*p* < 0.05). The lowest dose of CurDG (25 mg/kg) exhibited significant analgesia only after 9 days of treatment, whereas 50, 100, and 200 mg/kg of CurDG treatment showed significant analgesia even after 3 days of treatment (Figure 3A). Moreover, CurDG or vehicle had no effects on the PWL of the contralateral paw (Figure 3B). Over the entire time-course, the overall thermal hyperalgesia of CCI mice treated with CurDG was significantly improved compared to the vehicle-treated mice (*p* < 0.05), confirmed by the significantly higher AUC of CurDG-treated mice compared to the vehicle-treated mice (Figure 3C). In addition, though, pregabalin 40 mg/kg exhibited higher efficacy, 100 and 200 CurDG showed comparable anti-thermal hyperalgesic effect with pregabalin.

### 2.3. Motor Performance

**Figure 4:**
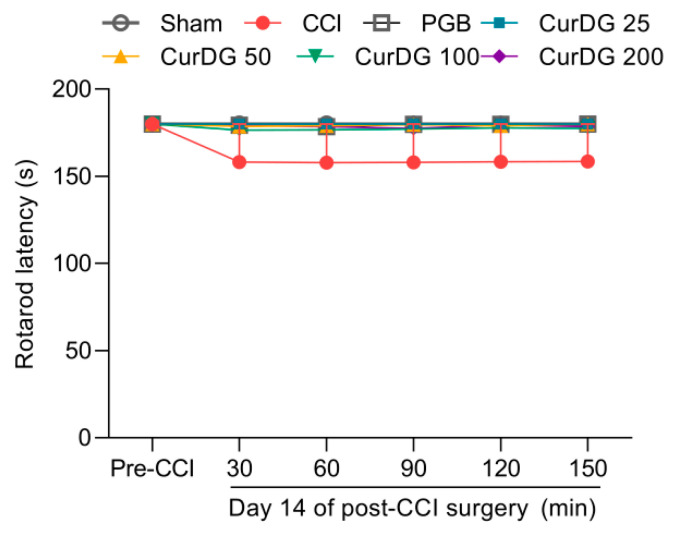
Effect of vehicle, pregabalin (PGB), and CurDG on the motor coordination in CCI-mice (Data are shown as mean ± SEM; *n* = 8 mice per group).

The rotarod test was conducted to rule out the possible nonspecific muscle relaxant effects from repeated CurDG treatment. As shown in Figure 4, treatment with CurDG or pregabalin had no significant effect on the balance and motor coordination throughout the 30–150 min time course compared to the vehicle-treated CCI mice.

### 2.4. CurDG Decreases TNF-α and IL-6 Expression in the Sciatic Nerve and Spinal Cord

**Figure 5:**
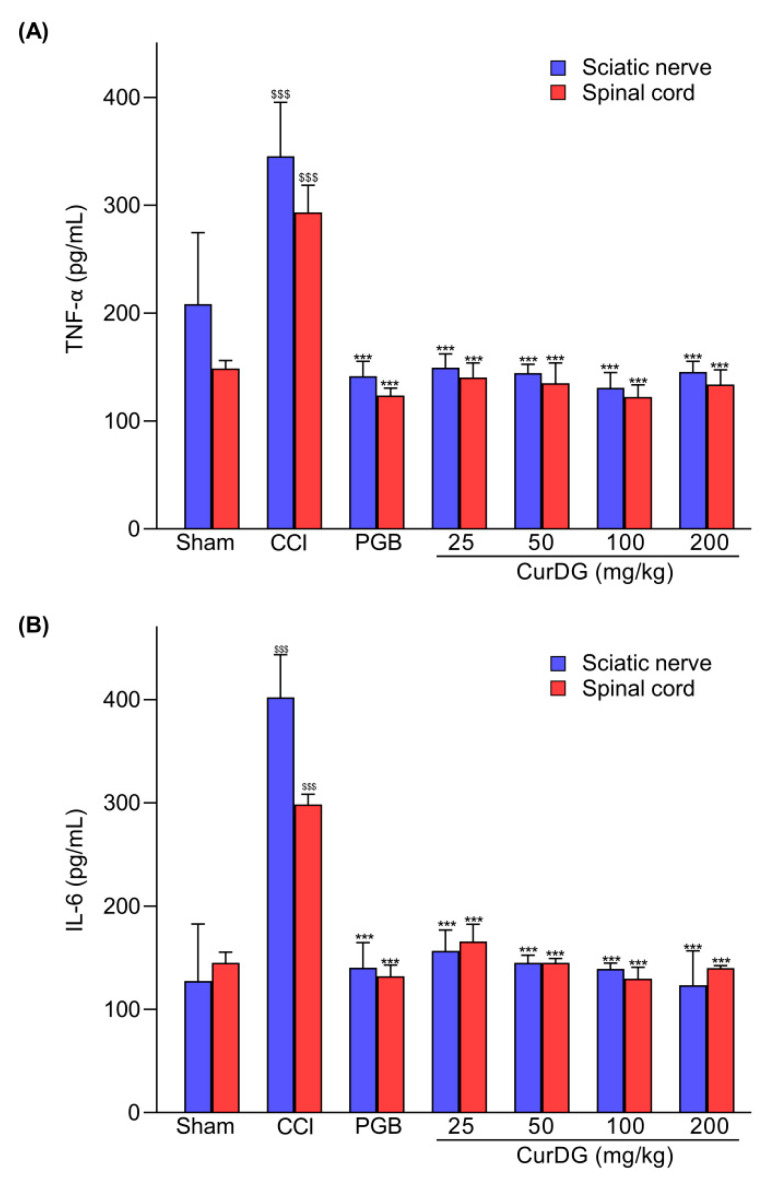
The increased TNF-α and IL-6 levels in CCI-mice were diminished after the administration of CurDG (25, 50, 100, 200 mg/kg). (**A**) TNFα and (**B**) IL-6 expression in both the spinal cord and sciatic nerve. (Means ± SEM; ^$$$^ and *** indicate the statistical significance at *p* < 0.001 compared to the sham group and the vehicle-treated groups, respectively).

To evaluate the effect of CurDG on known inflammatory processes, we measured the TNF-α and IL-6 levels in both the sciatic nerve and spinal cord. As shown in Figure 5, the expression of cytokines in the sciatic nerve and the spinal cord in the CCI-group increased significantly as expected in comparison to the sham control group (*p* < 0.001). The oral administration of CurDG diminished the overexpression of TNF-α (Figure 5A) and IL-6 (Figure 5B) in both the sciatic nerve and spinal cord of CCI-mice. With respect to pregabalin, CurDG at all doses tested produced comparable reduction in inflammatory mediators in both the spinal cord and sciatic nerve.

## 3. Discussion

**Figure 6:**
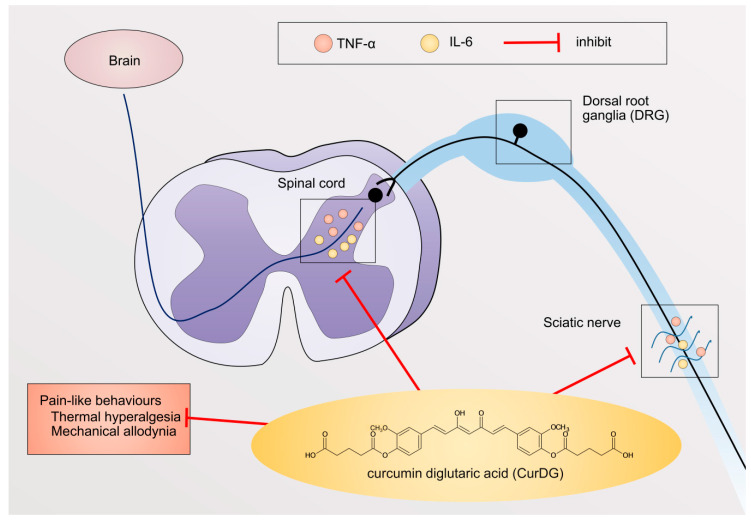
Systematic mechanism of action of CurDG. CurDG inhibits the expression of TNF-α and IL-6 in the sciatic nerve and spinal cord of the CCI-mice, as well as suppresses pain-like behaviors: thermal hyperalgesia and mechanical allodynia.

The pro-inflammatory cytokines, including TNFα and IL-6, have been reported to be involved in the pathogenesis of neuropathic pain. This is associated with the interactions of peripheral immune cells to peripheral nerves, as well as the interactions between resident immune cells in the spinal cord to the second-order neurons [4]. In turn, this causes increased pain-like behaviors, such as mechanical allodynia and thermal hyperalgesia [4,6]. Thus, studies reported that the inhibition of TNFα and IL-6 expression could prevent pain progression [27,28]. Accordingly, in the present study, we investigated the potential effect of CurDG in the CCI-induced neuropathic pain mice model. We demonstrated that CurDG alleviated the mechanical allodynia and thermal hyperalgesia in CCI mice without any effect on motor performance when compared to the vehicle-treated CCI mice. Moreover, the increased levels of TNF-α and IL-6 in both sciatic nerve and spinal cord following CCI were suppressed to the control level (sham group) in response to the CurDG treatment (Figure 6).

Due to the involvement of inflammation in the development of pain sensitization in neuropathic pain, many studies have been carried out using natural products possessing anti-inflammatory properties to identify potential therapeutic agents: e.g., rosmarinic acid, astaxanthin, and quercetin [29,30,31]. Clusters of evidence indicate the therapeutic effectiveness of curcumin in the management of neuropathic pain. Curcumin produces analgesia via different mechanisms, including the suppression of immune response, modulation of pain-associated neurotransmitters, and blockage of the transient receptor potential vanilloid type I (TRPV1) receptors [32,33,34,35]. The oral administration of curcumin at 50 mg/kg for 7 days was reported to produce a significant reduction in mechanical allodynia in CCI-rats [36]. Curcumin at 40, 50, 60, and 120 mg/kg significantly reduced thermal hyperalgesia and mechanical allodynia when administered intraperitoneally for 7 days via suppressing brain-derived neurotropic factor (BDNF), cyclooxygenase-2 (COX-2), and spinal interleukin 1 beta (IL-1β) [37]. In the present study, CurDG, at the lowest dose tested (equimolar to 25 mg/kg curcumin), demonstrated a significant reduction in both mechanical and thermal pain hypersensitivities compared to the CCI-control group (*p* < 0.05). Though many studies reported the baseline PWT of mice lying below 1.4 g, the baseline PWT value of ≈ 4 g, obtained in this study, was consistent with several previous studies [38,39]. Moreover, as per the AUC data obtained for the entire time-course, a dose-dependent reduction in both mechanical and thermal pain-hypersensitivities was observed with 25, 50, and 100 CurDG (*p* < 0.05), while 200 CurDG showed comparable analgesia with 100 CurDG. Hence, CurDG might have reached its maximum effect at a dose equimolar to 100 mg/kg curcumin.

As per the literature, studies conducted using increasing doses of curcumin demonstrated the ineffectiveness of curcumin in alleviating nerve injury-induced pain like behaviors at lower doses. For example, a study conducted by Xiaoyan Zhu et al. demonstrated no significant analgesic effect with 7 days administration of 20 mg/kg curcumin intraperitoneally (i.p.) in CCI rats [37], whereas Zanjani et al. demonstrated no significant analgesia with 12.5 and 25 mg/kg (i.p.) treatment for 7 days [32]. As a corollary, another study conducted in spared nerve-injured mice showed insignificant analgesia with the treatment of 30 mg/kg (i.p) curcumin for 7 days [40]. Hence, many studies used oral or i.p. dose of 50 mg/kg or 100 mg/kg curcumin as a single dose in the treatment of CCI-induced neuropathy in rodents [10,36,41,42]. Interestingly, in the present study, effective analgesia was obtained with the lowest tested dose of CurDG, which was equimolar to 25 mg/kg curcumin, as 25 CurDG produced significant analgesia at day 7 and 50 mg at day 3. This might result from the better physicochemical profile of CurDG [26] attributed to the reduction of the oral dose and, accordingly, the improved therapeutic effectiveness of CurDG compared to its parent drug, curcumin.

Currently, it has been recognized that the complex mechanisms involved in pain pathogenesis restrict the efficacy of neuropathic pain treatment. Immune responses in the peripheral nerves and spinal cord are identified as the major contributing factor for the development and maintenance of neuropathic pain [5]. This is characterized by the accumulation and infiltration of peripheral immune cells, such as T cells, macrophages, and mast cells, in the sciatic nerve and dorsal root ganglia (DRG) [4]. Besides, several inflammatory mediators are produced as a response to peripheral nerve injuries, such as TNF-α and IL-6 [6,43]. These pro-inflammatory cytokines released by the peripheral immune cells surrounding the nerve injury induces peripheral sensitization [4]. The cytokine TNF-α activates TNF receptors (TNFR) in peripheral nerves via p38 activation, leading to increased pain transduction. Akin to TNF-α, IL-6 also binds to its receptor, IL-6 receptor (IL-6R), and induces neuronal excitability of nociceptors [5]. Moreover, in peripheral nerve injury, IL-6, IL-6R, and gp130 (a signal transducer of IL6R) are overexpressed, making it possible to cause significant pain hypersensitivity [44]. In the present study, nerve injury of CCI-mice caused overexpression of both TNF-α and IL-6, as well as pain hypersensitivity. As anticipated, CurDG (25–200 mg/kg)-treated CCI-mice showed a reduction of TNF-α and IL6 levels in the sciatic nerve in parallel with decreasing hyperalgesia and allodynia, suggesting that the underlying mechanism of CurDG as an analgesic agent likely includes inhibition of TNF-α and IL-6 in the sciatic nerve.

In addition, the pro-inflammatory mediators produced as a response to peripheral nerve injury induce pain transmission to the brain cortex [6]. Peripheral nerve injury, i.e., CCI, can also activate resident spinal glia indicated by the excessive release of inflammatory mediators, leading to the sensitization of second-order neurons, known as central sensitization [4,5]. This activation is characterized by the increase of microglia marker (ionized calcium-binding adaptor molecule 1; iba1), astrocyte marker (glial fibrillary acidic protein; GFAP), as well as the release of pro-inflammatory mediators, including TNF-α and IL-6, produced by glial cells [45]. As reported, these cytokines involve spinal sensitization via enhancing excitatory neurotransmission and suppressing inhibitory neurotransmission [46]. Minocycline, a potent inhibitor of resident glia in the spinal cord, has been found to be effective in pain treatment in both preclinical and clinical trials, suggesting that targeting neuroinflammation in the spinal cord could be a promising therapeutic strategy [27,28]. In the spinal cord injury, curcumin has been found to improve responses to pain as observed by increased locomotor activity when compared to the control spinal cord injury group. In the same study, curcumin (100 mg/kg, i.p.) also reduced the expression of pro-inflammatory cytokines in the spinal cord, such as TNF-α, IL-1β, IL-6, via the nuclear factor kappa B (NF-κB) pathway [47]. Consistently, in mice with brachial plexus avulsion, curcumin inhibited pain-like behaviors, spinal glia activation, as well as pro-inflammatory cytokine levels, such as TNF-α and IL-6 [48]. In addition, the activity of curcumin on spinal neuroinflammation was observed in sickle mice, as evidenced by the suppression of glial activation, leading to decreasing thermal and mechanical hypersensitivities [49]. In the present study, the pro-inflammatory TNF-α and IL-6 were significantly increased in the spinal cord of CCI-mice, and repeated administration of CurDG reduced the increased TNF-α and IL-6 levels, returning to normal (sham), indicating the anti-inflammatory activity of CurDG, which was in line with curcumin. Therefore, the exerted reduction in pain hypersensitivity observed with CurDG likely involved curcumin by decreasing the pro-inflammatory cytokines TNF-α and IL-6 in both spinal neuroinflammation and peripheral inflammation.

In a clinical study conducted using patients with osteoarthritis, the analgesic efficacy of Flexofytol^®^, which is a new licensed formulation of curcumin with improved intestinal absorption, was investigated. The results showed the effectiveness of Flexofytol^®^ in improving patients’ pain level with the treatment of 4–6 capsules per day (each capsule contains 42 mg of optimized curcumin) along with a high degree of patient satisfaction while maintaining a favorable safety profile [50]. Therefore, it thus appears that curcumin prodrugs with improved pharmacokinetic profiles should be further studied as possible analgesic agents. In the present study, we demonstrated that CurDG significantly alleviated thermal and mechanical hypersensitivities while diminishing TNF-α and IL-6 expression in peripheral nerve and spinal cord of CCI-mice. This study, along with our previous study on the antinociceptive effects of CurDG, evidenced the potential of the curcumin prodrug, CurDG, as an analgesic agent of improved therapeutic efficacy.

## 4. Materials and Methods

### 4.1. Chemicals and Reagents

CurDG was synthesized by Natural Products for Aging and Chronic Diseases Research Unit, Faculty of Pharmaceutical Sciences, Chulalongkorn University, Bangkok, Thailand [26]. Carboxymethyl cellulose (CMC) and other chemicals were purchased from Sigma-Aldrich (St. Louis, MO, USA). Zoletil (tiletamine HCl and zolazepam HCl) was obtained from Virbac Laboratories (Carros, France). Septichlor (chlorhexidine 1.5% *w*/*v* & cetrimide 15% *w*/*v*) was purchased from New Life Pharma Co., Ltd. (Bangkok, Thailand). ELISA kits were purchased from BioLegend (San Diego, CA, USA).

### 4.2. Animals

Male Institute of Cancer Research (ICR) mice weighing 25–35 g were housed in a temperature-controlled room (24 ± 2 °C and 40–60% humidity) with a 12/12 h light-dark cycle with *ad libitum* access to food and water at the animal facility of the Faculty of Pharmaceutical Sciences, Chulalongkorn University. Animals were allowed to acclimate to the facility for a minimum of one week. All protocols were previously approved by the Institutional Animal Care and Use Committee (IACUC) of the Faculty of Pharmaceutical Sciences at Chulalongkorn University in Thailand (protocol number: 1833017).

### 4.3. Chronic Constriction Injury

A model of peripheral neuropathy was induced in mice by the method described previously [51]. Briefly, mice were anesthetized with Zoletil^®^ at 35–50 mg/kg, i.p, and the left sciatic nerve of the paw was exposed at mid-thigh level and tied loosely by three ligations with 1 mm spacing. For the mice in the sham group, the sciatic nerve was exposed without the ligations. Incisions were closed with sutures. After the surgery, all mice were observed closely until they recovered from anesthesia.

### 4.4. Treatments and Experimental Timeline

Animals were randomly divided into seven groups: the 2 groups of vehicle control (sham and CCI groups), pregabalin-treated group, and 4 groups of CurDG at the doses equimolar to 25, 50, 100, and 200 mg/kg/day curcumin (denoted as 25, 50, 100, and 200 CurDG). Pregabalin was suspended in 0.5% carboxymethyl cellulose (CMC) in normal saline and administered at 40 mg/kg. CurDG was suspended in 0.5% CMC in normal saline. All treatments were administered orally in the final volume of 10 mL/kg, once a day 24 h post-CCI-surgery for 14 days. One hour after treatment, the von Frey and plantar tests were performed on days 3, 5, 7, 9, 11, and 14 post-CCI. Further, the rotarod test was performed on day 14. At the end of the experiment, mice were euthanized, and the spinal cord and sciatic nerve were extracted (Figure 1B).

### 4.5. Von Frey Test

The anti-mechanical allodynic effect was evaluated by the von Frey test using a series of von Frey filaments (Stoelting Co., Wood Dale, IL, USA). Mice were placed into a Plexiglas chamber on an elevated wire mesh grid, and filaments were applied to the plantar surface of the paw in a series of ascending forces. Lifting, shaking, or licking the paw was considered as a positive response. The mechanical threshold (paw withdrawal threshold; PWT) was defined as the minimal force that induces at least three paw withdrawal reflexes observed out of five consecutive trials, which was expressed in grams (cut off = 5 g).

### 4.6. Plantar Test

The anti-thermal hyperalgesic effect of CurDG was evaluated by the plantar test using the Hargreaves apparatus (Ugo Basile, Italy). Mice were placed in a Plexiglas chamber with a clear platform, and the plantar surface of the mouse paw was exposed to a beam of infrared light. The paw withdrawal latencies (PWL) were recorded as their tolerance times from the initial application of the light beam to the elevation of the paw (cut off = 12 s).

### 4.7. Rotarod Test

The rotarod test was used to evaluate the effect of CurDG on balance and motor coordination as a sedative side effect using the Rotarod apparatus (Ugo Basile, Italy). Mice were placed on a constantly rotating bar (20 rpm) and trained to remain on the bar for 180 s. On the day 14 of CCI surgery, mice were administered their treatments, and the duration that mice were capable of remaining on the rod was measured at 30, 60, 90, 120, and 150 min after the treatment.

### 4.8. Tissue Collection and Analysis of Pro-Inflammatory Cytokines

Following euthanization on day 15 post-CCI surgery, spinal cords (lumbar 4–6) and ipsilateral sciatic nerve were collected and homogenized on ice and centrifuged at 12,000 *g* at 4 °C for 10 min. The supernatant was kept and stored at −20 °C. Two pro-inflammatory cytokines, TNF-α and IL-6, were measured in the collected supernatants of the spinal cord and sciatic nerves using commercially available ELISA assays (BioLegend, San Diego, CA, USA). Briefly, the supernatant was transferred to the ELISA plates after pre-coating with anti-mouse TNF-α and IL-6 antibodies. Then, the standard curve was used to determine the expression levels of TNF-α and IL-6 in the sciatic nerve and spinal cord.

### 4.9. Statistical Analysis

The animal results are presented as mean ± SEM, and the differences in the mean values between groups were analyzed using ANOVA, followed by Bonferroni post hoc test using GraphPad Prism version 8.0.2. Statistical significance was considered to be achieved when *p* was <0.05.

## 5. Conclusions

Our findings demonstrated the anti-allodynic and anti-hyperalgesic efficacy of CurDG as supporting pre-clinical evidence to develop CurDG as a novel analgesic drug for the treatment of neuropathic pain.

## Abbreviations

AUC	Area under the curve
BDNF	Brain-derived neurotrophic factor
CCI	Chronic constriction injury
CIPN	Chemotherapy-induced peripheral neuropathy
CMC	Carboxymethylcellulose
COX-2	Cyclooxygenase-2
CurDG	Curcumin diglutaric acid
DRG	Dorsal root ganglia
GFAP	Glial fibrillary acidic protein
Iba-1	Ionized calcium-binding adaptor molecule 1
ICR	Institute of Cancer Research
IL-6	Interleukin-6
IL-6R	IL-6 receptor
IACUC	Institutional Animal Care and Use Committee
i.p.	intraperitoneally
NF-κB	Nuclear factor kappa B
PWL	Paw withdrawal latency
PWT	Paw withdrawal threshold
SEM	Standard error of mean
TNF-α	Tumor necrosis factor alpha
TNFR	TNF receptors
TRPV1	Transient receptor potential vanilloid type I
